# The Genomic Response to TGF-β1 Dictates Failed Repair and Progression of Fibrotic Disease in the Obstructed Kidney

**DOI:** 10.3389/fcell.2021.678524

**Published:** 2021-07-02

**Authors:** Craig E. Higgins, Jiaqi Tang, Stephen P. Higgins, Cody C. Gifford, Badar M. Mian, David M. Jones, Wenzheng Zhang, Angelica Costello, David J. Conti, Rohan Samarakoon, Paul J. Higgins

**Affiliations:** ^1^Department of Regenerative and Cancer Cell Biology, Albany Medical College, Albany, NY, United States; ^2^The Urological Institute of Northeastern New York, Albany, NY, United States; ^3^Division of Urology, Department of Surgery, Albany Medical College, Albany, NY, United States; ^4^Department of Pathology and Laboratory Medicine, Albany Medical College, Albany, NY, United States; ^5^Division of Transplantation Surgery, Department of Surgery, Albany Medical College, Albany, NY, United States

**Keywords:** fibrosis, PAI-1, transcription, TGF-β, p53

## Abstract

Tubulointerstitial fibrosis is a common and diagnostic hallmark of a spectrum of chronic renal disorders. While the etiology varies as to the causative nature of the underlying pathology, persistent TGF-β1 signaling drives the relentless progression of renal fibrotic disease. TGF-β1 orchestrates the multifaceted program of kidney fibrogenesis involving proximal tubular dysfunction, failed epithelial recovery or re-differentiation, capillary collapse and subsequent interstitial fibrosis eventually leading to chronic and ultimately end-stage disease. An increasing complement of non-canonical elements function as co-factors in TGF-β1 signaling. p53 is a particularly prominent transcriptional co-regulator of several TGF-β1 fibrotic-response genes by complexing with TGF-β1 receptor-activated SMADs. This cooperative p53/TGF-β1 genomic cluster includes genes involved in cellular proliferative control, survival, apoptosis, senescence, and ECM remodeling. While the molecular basis for this co-dependency remains to be determined, a subset of TGF-β1-regulated genes possess both p53- and SMAD-binding motifs. Increases in p53 expression and phosphorylation, moreover, are evident in various forms of renal injury as well as kidney allograft rejection. Targeted reduction of p53 levels by pharmacologic and genetic approaches attenuates expression of the involved genes and mitigates the fibrotic response confirming a key role for p53 in renal disorders. This review focuses on mechanisms underlying TGF-β1-induced renal fibrosis largely in the context of ureteral obstruction, which mimics the pathophysiology of pediatric unilateral ureteropelvic junction obstruction, and the role of p53 as a transcriptional regulator within the TGF-β1 repertoire of fibrosis-promoting genes.

## The Clinical Realities of Chronic Renal Disease

**FIGURE 1:**
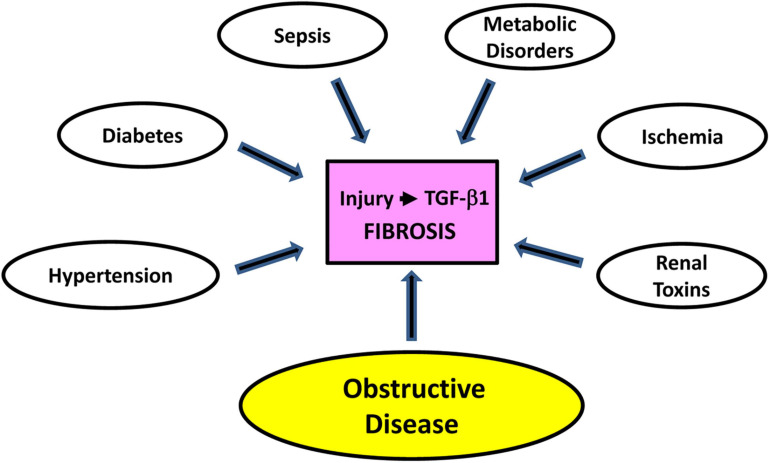
Contributors to renal damage. Extensive trauma or episodic epithelial injury, regardless of etiology and usually in the context of persistent transforming growth factor-β1 (TGF-β1) pathway activation, initiates and sustains a program of maladaptive repair that facilitates the progression of AKI to CKD. While diabetes and hypertension are preeminent initiators of CKD, sepsis, metabolic disorders, ischemia/reperfusion injury, exposure to nephrotoxins, and obstructive nephropathy are other significant causative factors. Several animal models of renal injury lend themselves to the discovery of genes and pathways that contribute to the onset and progression of kidney fibrosis. Unilateral ureteral obstruction (UUO) in rodents (either complete or partial/reversible), for example, is one of the most widely used as it approximates the pathophysiology of human obstructive nephropathy in children and adults. Ureteral ligation is a relatively simple procedure and produces a highly reproducible pathological response over a short time course with minimal inter-animal variability. UUO provides a translationally-relevant *in vivo* platform to probe the genomic complexity of kidney injury, mechanisms underlying maladaptive repair and the efficacy of new therapeutic approaches to the management of fibrotic disease (Martínez-Klimova et al., 2019).

Acute kidney injury (AKI) and chronic kidney disease (CKD) comprise a rapidly growing medical and economic burden within the US as well as globally. Renal tubular epithelial trauma and subsequent cell death correlates with patient morbidity and mortality and, when severe or episodic, often progresses to CKD and eventual end-stage renal disease (ESRD) (Bonventre and Yang, 2011; Kaissling et al., 2013; Ferenbach and Bonventre, 2015; Kumar, 2018; Liu et al., 2018). Epidemiologic data suggest that CKD may be the most under-recognized public health issue impacting 1 in 7 (35 million) adults in the US with 90% of affected individuals unaware of their underlying condition (Tuot et al., 2011; Centers for Disease Control and Prevention, 2016^1^; US Renal Data System, 2018). The Global Burden of Disease Study^2^ (Bowe et al., 2018; O’Brien, 2019) estimated that over the period from 2002 to 2016, deaths due to CKD rose 58%. Moreover, disability adjusted life years lost to CKD climbed 41% while years living with disability and years of life lost to CKD increased by 48 and 56%, respectively. Diabetes and hypertension are the primary and secondary drivers, respectively, of CDK and ESRD (NIDDK Health Information Website)^3^; other prominent contributors include sepsis, ischemia/reperfusion injury, obstructive nephropathy, metabolic disorders, and dietary exposure to nephrotoxins (Uchino et al., 2005; Bagshaw et al., 2008; Emlet et al., 2015; Figure 1). Medicare costs for patients with all stages of CKD approximated $114 billion in 2016 alone ($35 billion for ESRD and $79 billion for the treatment of individuals with CKD without end-stage organ failure). Race, age and economic disparities are prevalent in the renal disease patient population (Luyckx et al., 2018) and the overall incidence as well as expenditures continue to rise with limited effective therapies on the horizon (Ruiz-Ortega et al., 2020).

Regardless of etiology, progressive tubulointerstitial fibrosis is the final common pathway to CKD and a hallmark of ESRD (Eddy, 2005, 2014; Bonventre, 2010; Zeisberg and Neilson, 2010). Indeed, the extent of tubulointerstitial pathology (i.e., degree of inflammation, tubular dysmorphism and atrophy, progressive fibrosis) has important functional and prognostic implications (Grande et al., 2010; Truong et al., 2011; Eddy, 2014). While older individuals constitute the majority of the at-risk cohort (60% of the population >80 years have CKD), with collateral age-dependent increases in cardiovascular complications (Ruiz-Ortega et al., 2020), children are also susceptible. In 2016, approximately 5,700 pediatric patients developed ESRD due to several causative factors with a mortality incidence 30-times that of their healthy counterparts (McDonald et al., 2004; Kramer et al., 2009; Centers for Disease Control and Prevention, 2016; US Renal Data System, 2018). Indeed, the primary causes of pediatric CKD and ESRD are congenital anomalies of the kidney and urinary tract (Ingraham and McHugh, 2011; Chevalier, 2016). Unilateral ureteropelvic junction (UPJ) obstruction, with an incidence of 1:500–1,500 live births, is the most common form of obstructive uropathy associated with end-stage disease although other contributors include ureterovesical junction blockage, posterior urethral valve disease, urethral atresia or stricture and neuropathic bladder (Ucero et al., 2010; Weitz et al., 2017).

## Tubulointerstitial Injury: The Basics

**FIGURE 2:**
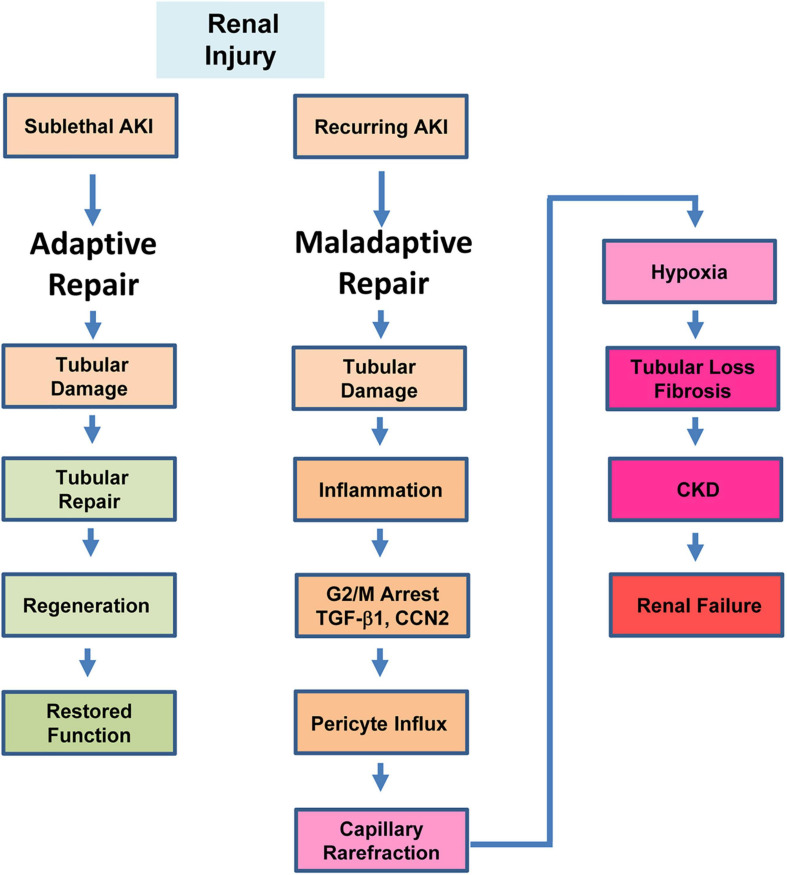
Repair outcomes in the injured kidney. Mild or sublethal AKI initiates a process of adaptive repair that involves resolution of inflammation and restoration of tubular architecture with a regain of renal function. Recurring AKI or severe tubular trauma, in contrast, results in maladaptive repair which is characterized by a sustained inflammatory response, stalling of injured proximal tubular epithelial cells in the G_2_/M stage of the cell cycle, interstitial translocation of vascular pericytes and their differentiation into ECM-producing myofibroblasts, tubular atrophy, capillary loss and failure to regenerate a functional epithelium. G_2_/M-stalled proximal tubular cells express significant levels of the potent pro-fibrotic effectors TGF-β1 and CCN2 which contribute to the initiation and progression of renal fibrosis.

Extensive or recurring sublethal epithelial trauma, usually in the context of persistent transforming growth factor-β1 (TGF-β1) pathway activation, initiates and sustains a program of maladaptive repair that facilitates the progression of AKI to CKD (Friedman et al., 2013; Emlet et al., 2015; Ferenbach and Bonventre, 2015; Venkatachalam et al., 2015; Basile et al., 2016; Takaori et al., 2016; Chang-Panesso and Humphreys, 2017; Schnaper, 2017; Chung et al., 2018; Qi and Yang, 2018; Gewin, 2019; Tang et al., 2020; Figure 2). The major source of TGF-β1, as well as other proinflammatory cytokines, in the kidney is the injured epithelium although both resident and infiltrative macrophages are also major contributors (Bonventre and Yang, 2011; Liu et al., 2018; Black et al., 2019; Zhang et al., 2020). Repetitive tubular damage triggers renal inflammation, pericyte loss, subsequent capillary rarefaction and tissue hypoxia, epithelial dedifferentiation, G_2_/M growth arrest, tubule dysfunction and nephron dropout (Basile, 2004; Fine and Norman, 2008; Yang et al., 2010; Moonen et al., 2018; Kumar, 2018; Zhang D. et al., 2018; Zhang S. et al., 2018; Liu et al., 2019). Necrotic or apoptotic renal epithelial cells also release various damage-associated molecular pattern (DAMP) factors that activate toll-like receptors and stimulate the innate immune system prolonging the inflammatory response (Liu et al., 2018).

Non-resolving inflammation precedes, and likely promotes, renal interstitial fibrosis (Bascands and Schanstra, 2005; Chevalier et al., 2010; Meng et al., 2015, 2016; Li et al., 2017). The extent of tubulointerstitial pathology (i.e., degree of inflammation, tubular dysmorphism and atrophy, progressive fibrosis) has critical functional and prognostic implications (Grande et al., 2010; Truong et al., 2011; Eddy, 2014). Increased angiotensin II and TGF-β1 levels in the injured kidney stimulates the conversion of activated Gli1^+^/FOXD1^+^ vascular pericytes and interstitial fibroblasts to matrix-producing myofibroblasts driving the pathophysiology of tissue fibrosis (Qi et al., 2006; Picard et al., 2008; Ricardo et al., 2008; Grande and Lopez-Novoa, 2009; Cook, 2010; Humphreys et al., 2010; LeBleu et al., 2013; Duffield, 2014; Gomez and Duffield, 2014; Kramann and Humphreys, 2014; Richter et al., 2015; Kramann et al., 2015; Mack and Yanagita, 2015). Pericyte mobilization in response to injury, moreover, results in their interstitial translocation, effectively promoting peritubular capillary collapse and creation of a hypoxic environment (Kramann et al., 2013; Kramann and Humphreys, 2014).

## Experimental Obstructive Nephropathy: A Tool to Probe Mechanisms and Pathways

Several animal models of acute and chronic renal disease are amenable to the discovery of causative factors underlying the onset and progression of kidney fibrosis while affording a platform to assess the efficacy of therapeutic interventions (Ortiz et al., 2015; Nogueira et al., 2017; Bao et al., 2018). Unilateral ureteral obstruction (UUO) in rodents (e.g., Chevalier, 2015; Martínez-Klimova et al., 2019), for example, closely mirrors (in an accelerated context) human obstructive nephropathy while bridging the pathologic features of AKI and CKD (Moller et al., 1984; Hruska, 2002; Ucero et al., 2014). Ureteral ligation provides an accessible, translationally-relevant, *in vivo* opportunity to clarify the genomic complexity of renal fibrotic disease, dissect critical pathophysiologic events underlying the kidney response to injury and identify mechanisms involved in maladaptive repair (Klahr and Morrissey, 2002; Truong et al., 2011; Eddy et al., 2012; Samarakoon et al., 2012; Arvaniti et al., 2016; Sun et al., 2016; Jackson L. et al., 2018; Jackson A. R. et al., 2018; Martínez-Klimova et al., 2019; Pavkovic et al., 2019).

Surgical interference with the flow of urine increases hydrostatic pressure initially in the collecting ducts expanding rapidly to the distal and proximal tubules (Martínez-Klimova et al., 2019). Long-term obstruction results in outer medullar ablation and tubular atrophy; a 65% decrease in proximal tubule mass becomes evident within 14 days of ureteral ligation. Tubule dilation, epithelial necrosis/apoptosis, basement membrane denudation, rapid influx of inflammatory cells, interstitial expansion with increased cellular proliferation and eventual fibrosis are prominent in the cortex of the ligated kidney (Cochrane et al., 2005; Manucha, 2007; Forbes et al., 2011, 2012; Ucero et al., 2014). The proximal tubule appears to be the predominant sensor and immediate effector of renal damage and may well orchestrate disease progression via injury-associated tubular shortening and/or paracrine mechanisms that impact several resident renal cell types (Endo et al., 2015; Tan et al., 2016; Gewin et al., 2017). A significant fraction (46%) of glomeruli, moreover, exhibit atrophic proximal tubules and 39% eventually become atubular indicating that the glomerulotubular junction tubular epithelium is particularly sensitive to UUO-induced necrosis and/or apoptosis (Chevalier et al., 2011). One suggestion is that glomerulotubular junction cell death may be a key driver of nephron loss and that the subsequent fibrotic response reflects an attempt at self-limiting tissue repair (Chevalier, 2016). Such congenital reduction in nephron density impairs recovery from obstructive injury and exacerbates the fibrotic process (Sergio et al., 2015).

Partial and complete UUO in neonatal rodents are similar except for a temporal offset in acquisition of pathologic features (Jackson L. et al., 2018; Jackson A. R. et al., 2018). UUO modeling largely focuses on the proximal tubular compartment due to its high mitochondrial load, dependency on oxidative phosphorylation, susceptibility to ischemic injury and relative deficiency of anti-oxidant/anti-apoptotic factors (Chevalier, 2016). The distal nephron including the collecting duct, however, also contributes significantly to the overall response of the kidney to ureteral ligation-induced injury (Hiatt et al., 2013). Tubular dilation and myofibroblast accumulation in the distal nephron increases by 2—3- and 6-fold, respectively, in the obstructed kidney and coupled to a change in cellular composition of the collecting duct. Aquaporin 2 (Aqp2)-expressing principal cells decline by 65% and intercalated cell abundance decreases by 75%. E-cadherin- and β-catenin-mediated collecting duct epithelial adhesion is also disrupted. Notably, these features are replicated in the distal and connecting tubules (Hiatt et al., 2013) confirming that the distal nephron is a major target of UUO-initiated renal disease, highlighting the utility of UUO as a model to dissect the involvement of collecting duct and distal tubule injury to kidney repair and fibrosis. Principal cells in the collecting duct are fundamental to the development of tubulointerstitial fibrosis (Butt et al., 2007; Ivanova et al., 2008; Fujiu et al., 2011), at least in part through Notch signaling, and are subject to epigenetic regulation (Zhang et al., 2020). Mib1, an E3 ligase produced by ligand-expressing cells, is required for efficient Notch mobilization while inactivation of Mib1 in the collecting duct results in increased tubulointerstitial fibrosis and apoptosis of principal cells in response to UUO. Furthermore, CKD can be induced by connecting tubule/collecting duct-specific disruption of the β1 integrin (Mamuya et al., 2017), integrin-linked kinase (Huang et al., 2019), and histone H_3_ K79 methyltransferase Dot1l (Zhang et al., 2020) or ameliorated by collecting duct-specific ablation of Krüppel-like factor 5 (Fujiu et al., 2011).

Recent genetic studies, moreover, implicate connecting tubule/connecting duct endothelin-1, a potent vasoconstrictor with proinflammatory and profibrotic properties, in not only UUO-mediated injury but also in streptozotocin-induced as well as age-related kidney disease (Zhang et al., 2020). Four groups of engineered mice including (1) those with floxed alleles of histone H_3_ lysine79 (H_3_K79) methyltransferase disruptor of telomeric silencing-1 (*Dot1l*^*f/f*^) and endothelin-1 (*Edn1*^*f*/f^); (2) *Dot1l*^*f/f*^ *Aqp2Cre* (*Dot1l*^*AC*^); (3) *Dot1l*^*f/f*^ *Edn1*^*f*/f^ *Aqp2Cre* (*DE*^*AC*^)*;* and (4) *Edn1*^*f*/f^ *Aqp2Cre* (*Edn1*^*AC*^) were subjected to UUO. An Aqp2 promoter-driven Cre construct provided for Cre expression specifically in the epithelial cells of the collecting duct. *Dot1l*^*AC*^ vs. WT or *Edn1*^*AC*^ mice developed severe fibrosis and renal dysfunction. *Dot1l*^*AC*^ phenotypes were mitigated in the double-knockout *DE*^*AC*^ mice with similar results evident in streptozotocin-induced diabetes and normal aging (Zhang et al., 2020). This is the first demonstration that loss of histone H_3_ K79 methyltransferase Dot1l promotes renal fibrosis due, in large measure, to endothelin-1 up-regulation in the collecting duct epithelium consistent with the implication that Dot1l exerts an antifibrotic function by repressing endothelin-1 transcription. Kidney fibrosis in response to UUO, moreover, is epigenetically regulated through Dot1l action in the connecting tubule and collecting duct. It appears, therefore, that the pathophysiology of obstructive uropathy is both complex and likely involves the entire nephron. The growing appreciation for the extensive cross-talk and mutual inducibility between the TGF-β1 and endothelin-1 signaling systems in the kidney, their shared potent fibrogenic activities and ability to impact virtually all renal cell types (e.g., Eddy, 2000; Castañares et al., 2007; Dhaun et al., 2012; Wermuth et al., 2016) suggests that nephron segment-specific fibrotic factors may need to be considered in the formulation of targeted therapies.

## Tubular Repair and Cell Cycle Arrest in the Injured Kidney

Depending on the severity and duration of injury to the proximal tubular epithelium (a critical initiator of the tubulointerstitial fibrotic process), the response of the kidney can be adaptive (i.e., regenerative; restoration of function) or maladaptive (i.e., fibrotic; compromised function) (Grgic et al., 2012; Lee et al., 2012; Kumar et al., 2014; Ferenbach and Bonventre, 2015; Kumar, 2018; Liu et al., 2018; Qi and Yang, 2018; Figure 2). Following tubular cell necrosis or apoptosis, the remaining viable epithelium undergoes morphologic dedifferentiation (i.e., loss of polarity with cell spreading and migration to cover the exposed areas of the basement membrane) and subsequent proliferation as an attempt to restore the functional integrity of the nephron (Bonventre, 2003). Fate mapping studies indicate, moreover, that tubular regeneration is orchestrated by surviving epithelial cells (Humphreys et al., 2008; Berger et al., 2014; Lombardi et al., 2016). Although it is apparent that upon injury a subpopulation of renal cells exhibits significant regenerative potential, these are not likely a fixed pre-existing progenitor population but rather derive from viable dedifferentiated proximal tubular cells that acquire a specific phenotype in response to injury (Kusaba and Humphreys, 2014; Kusaba et al., 2014; Humphreys et al., 2016; Andrianova et al., 2019). Early successful repair, nevertheless, involves activation of a Sox^+^/KIM1^+^ cohort which regresses after regeneration of a functional epithelium (Kumar, 2018). Retention of the Sox^+^/KIM1^+^ phenotype, however, signals tubules with unresolved injury while *Snai1* and *Twist1* induction predispose to a more plastic phenotype, failed differentiation and accumulation of cells in G_2_/M with engagement of a proinflammatory/profibrotic genomic program (Kumar, 2018).

**FIGURE 3:**
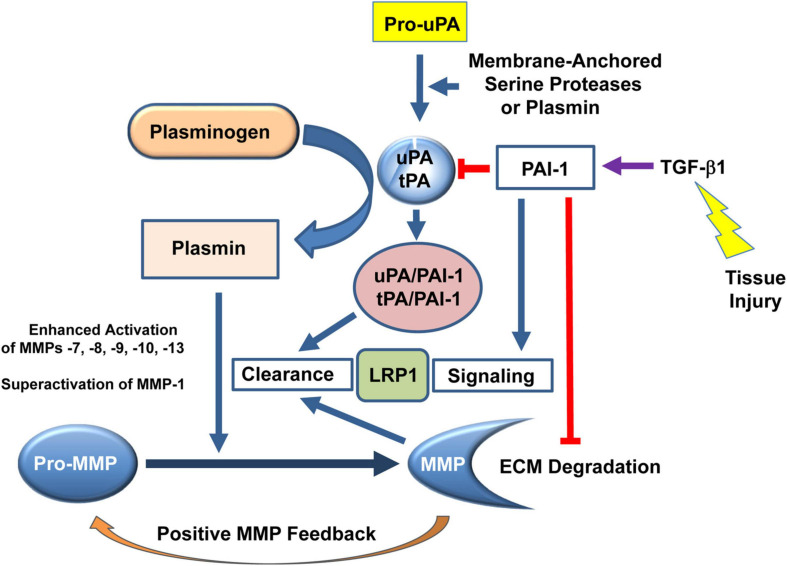
PAI-1 (SERPINE1) is a critical factor in the regulation of the pericellular proteolytic microenvironment and fibrotic response to tissue injury. Plasminogen activators (urokinase, uPA; tissue-type, tPA) are the physiologically and pathophysiologically-relevant plasmin-generating proteinases that impact extracellular matrix (ECM) accumulation/degradation through a complex and highly interdependent proteolytic cascade. Pro-uPA is cleaved to the active enzyme uPA by membrane-anchored serine proteases (e.g., Matriptase, Hepsin, Serase-1B) or catalytically-active levels of plasmin. uPA-induced conversion of plasminogen to plasmin results in the significant downstream mobilization of several matrix metalloproteinases (MMPs). Collectively, both the plasmin-dependent and MMP proteolytic systems dictate the extent and locale of ECM remodeling. Elevated expression or bioactivity of PAI-1, generally in response to tissue injury-induced TGF-β1, facilitates ECM accumulation and inhibits ECM degradation which, if prolonged or chronic, leads to the initiation and progression of fibrotic disease.

**FIGURE 4:**
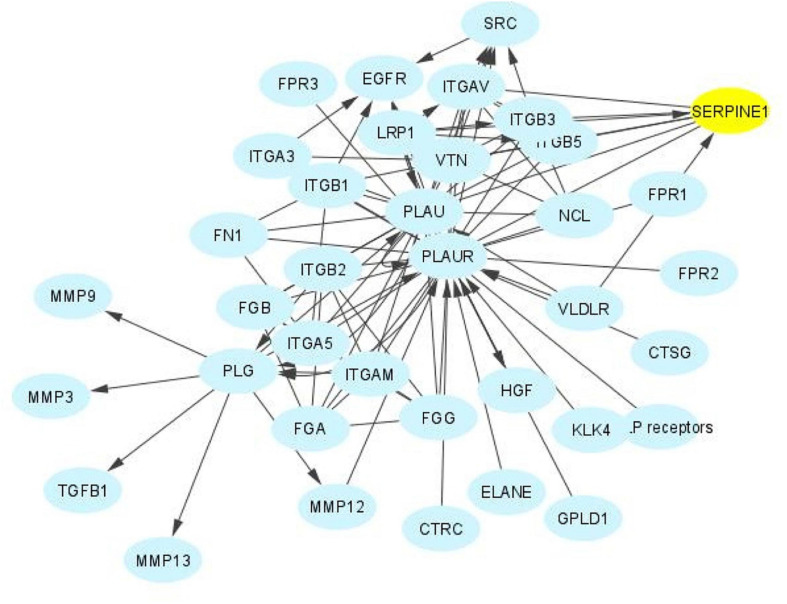
The SERPINE1 interactome. SERPINE1 (PAI-1) is a major hub factor in the regulation of the immediate pericellular proteolytic cascade. PAI-1 titrates the conversion of plasminogen to plasmin by binding to and inhibiting the catalytic activity of urokinase plasminogen activator (PLAU), effectively attenuating stromal proteolysis while promoting matrix accumulation and the onset and progression of fibrotic disease regardless of etiology. PAI-1 also regulates cellular attachment and migration, key aspects of the injury repair program, largely by altering interaction of the PLAU-PLAU receptor (PLAUR) complex with its associated integrins and by functioning as a ligand for LRP1 to initiate post-receptor downstream signaling.

**FIGURE 5:**
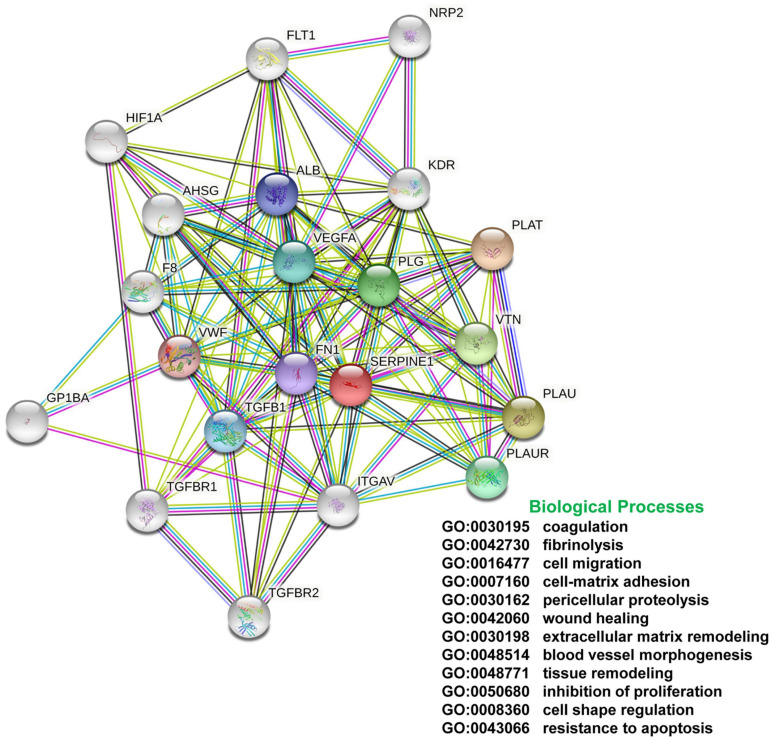
String Network and Gene Ontology. Protein-protein interaction and GO analyses of the SERPINE1/TGF-β1/fibronectin axis indicates that SERPINE1 is a significant nodal contributor to various biological processes that impact the global program of normal and maladaptive tissue repair. These data underscore the potential clinical utility of SERPINE1 targeting in the therapy of fibrotic disease.

G_1_ phase arrest in the injured kidney allows for repair of DNA damage prior to replication in S phase. G_2_/M-stalling provides an additional opportunity to assess DNA integrity but also mobilizes the c-JUN N-terminal kinase stress pathway resulting in the transcription of several major pro-fibrotic senescence-associated secretory phenotype (SASP)-type effectors. These include connective tissue growth factor (CTGF, CCN2), TGF-β1 and the clade E member 1 serine protease inhibitor SERPINE1, also known as plasminogen activator inhibitor-1 (PAI-1), a potent negative regulator of the pericellular proteolytic cascade (Yang et al., 2010; Sturmlechner et al., 2017; Liu et al., 2019; Figure 3). Cytoscape profiling, moreover, implicates SERPINE1 as a major hub gene in the genomic program of tissue fibrosis where it functions as a key interacting modulator of focalized uPA/uPAR-dependent pericellular proteolysis as well as a binding partner and activator of the signaling competent low-density lipoprotein receptor-related protein-1 (LRP1) (Figure 4). String Protein-Protein Interaction Network and Gene Ontology analyses confirmed the cooperative role of SERPINE1, TGF-β1 and the extracellular matrix (ECM) protein fibronectin in the more global process of normal and maladaptive wound repair (Figure 5).

Events underlying the coupling of G_2_/M and expression of a fibrotic program, however, are complex. TGF-β1-induced G_2_ phase prolongation in proximal tubular cells appears mediated, at least in part, by *Twist1* and *Snai1* since overexpression of either is sufficient for induction of the p53 target gene p21 and protracted residence in G_2_ (Lovisa et al., 2015; Qi and Yang, 2018). p21, moreover, is likely involved in the increase in G_2_ cells in the very initial stages of renal injury (Koyano et al., 2019). While the p53→p21 pathway contributes to G_2_/M arrest and acquisition of a fibrotic program, an additional highly up-regulated p53-dependent gene (at least in aristolochic acid [AA]-induced kidney injury) is cyclin G_1_ which promotes the extended duration of G_2_/M and also increases formation of target of rapamycin (TOR)-autophagy spatial coupling components (TASCCs) stimulating, thereby, expression of SASP genes (Canaud et al., 2019). The p53 inhibitor pifithrin-α (PIF) attenuates the fraction of G_2_/M-arrested epithelial cells while deletion of cyclin G_1_, mTOR, LC3, or lysosomal associated membrane protein 2 (LAMP2) reduces the onset and progression of renal disease (Canaud et al., 2019).

## Injury-Associated Acquisition of a Senescence-Like Phenotype

Multiple sublethal injuries to the kidney leads to the emergence of a senescence-like state in some surviving tubular cells resulting in a failure to respond with adaptive proliferation (Ferenbach and Bonventre, 2015). Senescent epithelial cells are evident in the kidney in the pathologic context of hypertension, diabetes, IgA nephropathy and ischemia/reperfusion injury particularly in aged mice, where progressive immune system dysfunction may drive the development of CKD (Verzola et al., 2008; Satriano et al., 2010; Qi and Yang, 2018; Xiong and Zhou, 2019; Schroth et al., 2020). Indeed, aging in rodents is associated with enhanced tubular cell senescence, elevated TGF-β1, p16, and p21 expression and increasing tubulointerstitial fibrosis (Ding et al., 2001; Knoppert et al., 2019). While reparative CD24^+^/CD133^+^ epithelial cells contribute to healing and functional recovery, exogenous delivery of even a small number of senescent cells induces inflammation and fibrosis (Kim et al., 2020).

**FIGURE 6:**
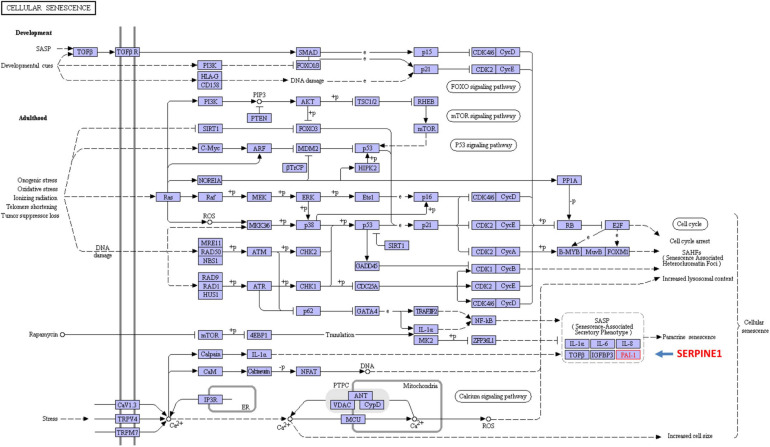
KEGG analysis of the highly interactive program of cellular senescence. SERPINE1 and TGF-β1, two prominent activators and members of the stress-activated SASP, are key factors in the global and renal programs of proliferative arrest. Several of the involved networks include the p53, *ras* and TGF-β signaling pathways. Collectively, these regulate the expression of a spectrum of cell cycle and growth control elements (e.g., p16, p21, TGF-β1, PAI-1) (Kortlever et al., 2006, 2008).

The maladaptive tubular repair and the cellular senescence programs (e.g., G_2_/M stalling, expression of proinflammatory/profibrotic factors) both involve p53 and transcription of the p53 target genes p21 and PAI-1. There is, in fact, considerable overlap among the SASP, the chronic kidney disease-associated secretory phenotype (CASP) and the SASP aging and disease biomarker gene sets that includes increases in the scar-promoting proteins TGF-β1, PAI-1 (SERPINE1) and CNN2 (Wang et al., 2017; Basisty et al., 2020). A percentage of tubular epithelial cells gradually acquire a senescence-like phenotype with advancing age and express elevated levels of TGF-β1, p16, and p21 (Ding et al., 2001; Braun et al., 2012; Ferenbach and Bonventre, 2015). Indeed, senescence promotes interstitial fibrosis, tubular atrophy and renal graft deterioration limiting tubular regeneration and transplant survival (Braun et al., 2012). The elevated levels of reactive oxygen species (ROS) that accompany the DNA damage response, moreover, are likely major contributors to the initiation of the senescent phenotype (Moonen et al., 2018; Beck et al., 2020a,b). Indeed, in some cell types, TGF-β1 functions as a senescence driver via ROS-stimulated NF-κB signaling and induction of SASP factors, including PAI-1 (Kwon et al., 2017; You et al., 2019; Figure 6). This appears critically important in the establishment of the growth arrest state as PAI-1 is not merely a biomarker of the senescent phenotype but is necessary and sufficient for the induction of replicative senescence downstream of p53 (Kortlever et al., 2006; Hiebert et al., 2018).

Once renal repair becomes dysfunctional (i.e., elevated expression of the cell cycle arrest protein p21, down-regulation of the anti-aging factor Klotho, telomere shortening, increased oxidative stress), continued activation of the SASP and CASP programs accelerate cellular aging leading to the development of age-related pathologies (Wang et al., 2017; Andrade et al., 2018; Dai et al., 2019). Cellular senescence is evident in many forms of kidney injury (Li and Lerman, 2020) and older mice have increased senescence-associated β-galactosidase, p53, and p21 expression in response to ischemia/reperfusion injury compared to young mice (Clements et al., 2013; Valentijn et al., 2018). This is relevant to the human condition as age-associated renal scarring, and decline in kidney function, varies among ethnic groups and expression of β-galactosidase and p16 is evident even in the absence of morphologic changes (Yang and Fogo, 2010).

Although the mechanism underlying cell cycle phase-specific arrest or at least residence prolongation is unclear, activation of the p53→p21 axis, particularly in the early stages of kidney disease, likely drives renal cell stalling in both G_1_ and G_2_/M phases (Yang et al., 2010; Overstreet et al., 2014; Moonen et al., 2018; Wu and Prives, 2018; Liu et al., 2019). In this regard, fibrosis in response to chemotherapeutic agents, nephrotoxins, ischemia/reperfusion injury or UUO is associated with DNA damage and normal aging sensitizes tubular epithelial cells to DNA damage-induced G_2_/M arrest (Yang and Fogo, 2010; Liu et al., 2018). Ataxia telangiectasia mutated (ATM) and ATM and RAD3-related (ATR), which function as sensors of DNA damage in the maintenance of genomic stability, are involved and alterations in their expression has consequences. ATM and ATR have several DNA repair targets in common including p53 and the cell cycle checkpoint kinases CHK1 (ATR) and CHK2 (ATM) (Bradbury and Jackson, 2003; Awasthi et al., 2015). ATR deletion in renal proximal tubular epithelial cells exacerbates maladaptive repair, increases the number of senescent cells and promotes expression of a profibrotic secretory phenotype (Kishi et al., 2019). These findings suggest that ATR provides a protective role in the injured proximal tubular epithelium to restrict or attenuate exuberant (i.e., fibrotic) repair while highlighting the role of p53 in renal disease since treatment with the p53 inhibitor PIF-α significantly reduces the fraction of G_2_/M cells and mitigates the fibrotic response (Yang et al., 2010; Overstreet et al., 2014; Liu et al., 2019).

**FIGURE 7:**
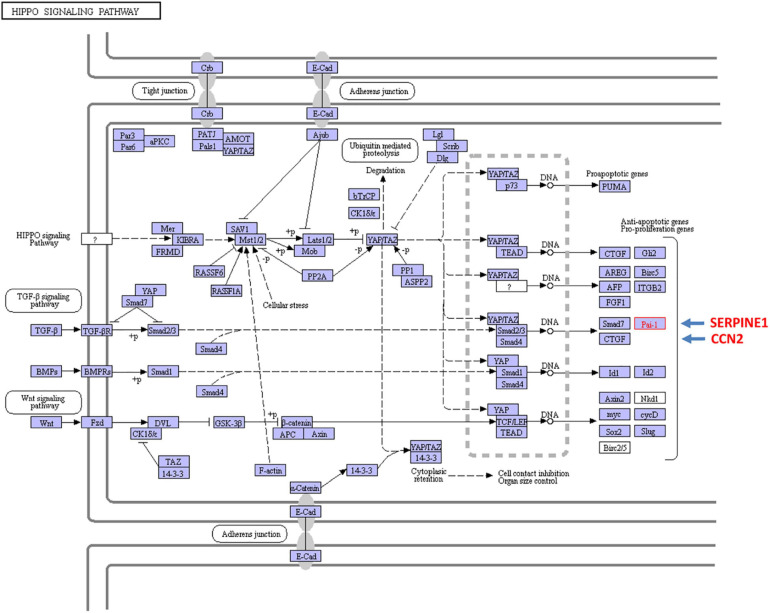
Cross-talk between the TGF-β and the YAP/TAZ pathways impact expression of the profibrotic SERPINE1 and CCN2 genes. TGF-β activates a canonical signaling network that involves the SMAD2/3-dependent transcription of SERPINE1 and CCN2. It is also evident that non-canonical pathway engagement (e.g., Hippo) contributes to maximal TGF-β1 SERPINE1 (PAI-1) and CCN2 (CTGF) expression by stimulating YAP/TAZ nuclear translocation and interaction with the TGF-βR-phosphorylated SMAD2/3 transcriptional effectors and the shuttle SMAD4.

Expression of a subset of TGF-β1 target genes that contribute to growth arrest, and G_2_/M stalling as well, appears to require both canonical and non-canonical signaling. To this point, TGF-β1 also upregulates the Hippo pathway effectors YAP (*yes*-associated protein) and TAZ (transcriptional co-activator with PDZ-binding motif) in proximal tubular epithelial cells both *in vivo* and *in vitro*. Indeed, doxycycline-induced tubular-specific TGF-β1 expression in double-transgenic Pax8-rtTA-tet-*o*-TGF-β1 mice enhances renal TAZ levels while TGF-β1 increases TAZ levels in human proximal tubular epithelial cells; *in vitro* modeling confirmed that TAZ is necessary for TGF-β1-mediated fibrogenesis (Anorga et al., 2018). Vector-driven TAZ synthesis in human proximal tubular (HK-2) cells, or addition of conditioned medium from TAZ overproducers to control vector-transduced HK-2 cells, mimics certain aspects of the TGF-β1-induced phenotype including G_2_/M arrest and acquisition of a profibrotic program (Anorga et al., 2018). Exposure of HK2 cells to hypoxic stress similarly promotes G_2_/M stalling and PAI-1 induction while TAZ overexpression leads to the accumulation of HK-2 cells in G_2_/M phase. TAZ is, in fact, required for maximal TGF-β1-mediated PAI-1 synthesis in proximal tubular cells (Liu et al., 2015; Samarakoon et al., 2015; Anorga et al., 2018; Bessho et al., 2019) and a similar involvement of YAP in TGF-β1-induced PAI-1 expression is evident in lung tumor cells (Kong et al., 2021). KEGG analysis confirmed that convergence of the TGF-β and Hippo signaling pathways regulates transcription of the profibrotic CCN2 and SERPINE1 genes (Figure 7). YAP knockdown, moreover, reduces levels of both CTGF (CCN2) and PAI-1 (SERPINE1) while introduction of the constitutively-active YAP^S127A^ construct increased PAI-1 expression (Marquard et al., 2020). Although the underlying mechanisms remain to be determined, YAP/TAZ apparently do not alter the rate of SMAD nuclear import or exit nor impact SMAD phosphorylation but may regulate SMAD nuclear levels by functioning, directly or indirectly, as retention factors and/or by changing TGF-βR activity (Labibi et al., 2020).

## TGF-β/SMAD Signaling Drives Fibrosis in Obstructive Nephropathy

**FIGURE 8:**
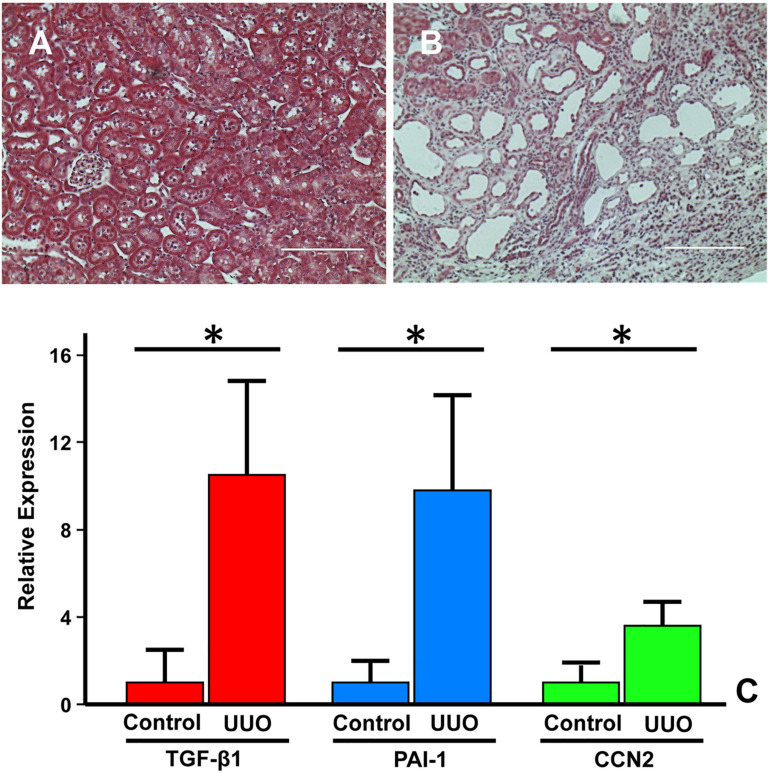
Fibrotic response of the murine kidney to UUO. Compared to the relatively normal histology of the contralateral control or sham-operated kidney **(A)**, a dysmorphic and flattened epithelium with extensive tubular dilation, expanses of denuded basement membrane and accumulation of connective tissue (blue stain) in the expanded interstitial regions is evident in the obstructed kidney **(B)**. **(A,B)**, Trichrome stain. Morphometric analyses of immunohistochemical-stained paraffin-embedded sections of the UUO-injured kidney and the contralateral control 7–14 days post-surgery, revealed significant increases in PAI-1, TGF-β1, and CCN2 in the obstructed kidney **(C)**. Histograms illustrate the mean ± SD staining intensity (ImageJ threshold analysis) for TGF-β1, PAI-1, and CCN2 between the two experimental groups. *p < 0.05.

Increased expression of the potent profibrotic cytokine TGF-β1 and the type I/II TGF-β1 receptors is a hallmark feature of virtually all forms of CKD (Böttinger, 2007). Tubulointerstitial pathology following experimental UUO appears largely due to elevated levels of TGF-β1, SERPINE1 and CCN2 in the injured kidney (Figure 8) mimicking the increased TGF-β1 expression in children with UPJ obstruction (Miyajima et al., 2000; Inazaki et al., 2004; Ucero et al., 2010). Within hours, the occluded kidney exhibits changes in hydrostatic forces and increased oxidative stress (Schreiner et al., 1988; Klahr and Morrissey, 2002; Dendooven et al., 2011). Tubular stretch further stimulates TGF-β1 expression (>20-fold), increases the epithelial apoptotic index, and leads to the development of an interstitial inflammatory infiltrate (Miyajima et al., 2000; Rohatgi and Flores, 2010). Persistently elevated renal TGF-β1 expression, even after relief of UUO (depending on the duration of obstruction and extent of pathology) frequently leads to progressive tissue injury, impaired regenerative growth, and eventual loss of organ function (Chevalier, 1999; Chevalier et al., 2009, 2010).

TGF-β1 mRNA levels steadily increase in several nephron segments as early as day-1 post-UUO followed by TGF-β1 protein upregulation (Isaka et al., 2000; Miyajima et al., 2000; Klahr and Morrissey, 2002; Yang et al., 2010; Makitani et al., 2020). TGF-β1 transcripts are most prominent in the tubular epithelia and, to a lesser extent, in a fraction of infiltrating macrophages (Kaneto et al., 1993; Fukuda et al., 2001). Attenuation of UUO-induced fibrosis upon administration of the anti-TGF-β antibody 1D11 or the TGF-β activin-like kinase 5 (ALK5) receptor signaling inhibitor SB-525334 further highlight involvement of the TGF-β pathway in ureteral obstruction-initiated renal scarring (Richards et al., 2018). The contribution of TGF-β1 to the fibrotic response, importantly, was confirmed using genetic approaches. Conditional overexpression of TGF-β1 in the tubular epithelium of Pax8-rtTA-tet-o-TGF-β1 double transgenic mice induces extensive peritubular fibrosis, focal nephron degeneration (Traykova-Brauch et al., 2008; Koesters et al., 2010) and TGF-β1-dependent loss of the SMAD phosphatase PPM1A (Tang et al., 2020). Similarly, Pax8 promoter-driven expression of a ligand-independent constitutively-active TGF-β type I receptor results in the acquisition of features typical of AKI (e.g., epithelial apoptosis, necrosis and dedifferentiation; renal inflammation) (Gentle et al., 2013). The albumin/TGF-β1 transgenic mouse (Kopp et al., 1996), moreover, recapitulates the pathophysiologic heterogeneity of CKD progression highlighting their utility in the discovery of disease progression signatures (Ju et al., 2009).

Elevated levels of TGF-β1 in the injured kidney direct the myofibroblastic differentiation of recruited vascular pericytes and resident fibroblasts while driving a program of pathologic ECM synthesis and advancing fibrosis (Bonventre, 2010; Meng et al., 2015, 2016; Sun et al., 2016; Chen et al., 2018; Feng et al., 2018; Higgins et al., 2018). Genetic deficiency of SMAD3, a major profibrotic effector of TGF-β1 signaling, or administration of the SMAD3 inhibitor SIS3 immediately after ureteral ligation, attenuates myofibroblast accumulation while suppressing deposition of collagen I and fibronectin (Sato et al., 2003; Inazaki et al., 2004; Zhang D. et al., 2018; Zhang S. et al., 2018). One mechanism may involve the SMAD3-dependent autoinduction of TGF-β1 by UUO-stimulated TGF-β1 expression (Sato et al., 2003). This has potential clinical ramifications since post-injury treatment with SIS3 also blunted the subsequent fibrotic response (Zhang D. et al., 2018; Zhang S. et al., 2018) suggesting that blockade of TGF-β1→ALK5 signaling to SMAD3 has therapeutic implications. Several pre-clinical studies, in fact, targeted SMAD3 as one modality for the treatment of UUO-induced renal disease (e.g., Li et al., 2010; Ji et al., 2018; Wang et al., 2018).

Initial observations did, in fact, support the premise that interstitial fibrosis and disease progression in the obstructed kidney can be mitigated by blockade of TGF-β1 expression or function via antisense phosphorothioate oligodeoxynucleotides, small interfering RNA (siRNA) or neutralizing antibodies (Isaka et al., 2000; Miyajima et al., 2000; Gagliardini and Benigni, 2006; Hwang et al., 2006). Overexpression of the latent form of TGF-β1, to minimize availability of active TGF-β1 in the tissue microenvironment, decreases the incidence α-smooth muscle actin-positive cells (presumably myofibroblasts) in the UUO-injured kidney and blocks SMAD2/3 activation (Huang et al., 2006, 2008). The peroxisome proliferator-activated receptor gamma agonist troglitazone similarly reduces development of UUO-induced renal interstitial fibrosis and inflammation through suppression of TGF-β1 expression (Kawai et al., 2009). Collectively, these data are consistent with the concept that TGF-β1 is, indeed, the key driver of fibrosis in UUO either directly by impacting the transcription of disease-relevant genes or indirectly via angiotensin signaling (Ishidoya et al., 1995; Pimentel et al., 1995; Fern et al., 1999; Satoh et al., 2001; Inazaki et al., 2004; Shin et al., 2005). Indeed, angiotensin stimulates the expression of ECM structural elements (e.g., collagen, fibronectin, laminin) as well as inhibitors of ECM degradation including PAI-1 (SERPINE1) through TGF-β1-dependent mechanisms, thus promoting tissue fibrogenesis (Kagami et al., 1994; Wolf, 2006). While global TGF-β1-null mice exhibit no gross abnormalities at birth but die soon thereafter due to wasting associated with severe multifocal inflammation (Yaswen et al., 1996), carefully focused anti-TGF-β therapies, and perhaps targeting disease-critical downstream genes or enhancers of TGF-β1 profibrotic signaling, may be a more prudent and translationally-adaptable therapeutic approach. As one example, small molecule (SK-216) pharmacologic inhibition of the activity of the TGF-β1 target PAI-1 attenuates TGF-β1-induced fibroblast to myofibroblast transition and lung fibrosis (Omori et al., 2016). Varga and Pasche (2009) suggest, moreover, that neutralizing antibodies, pathway antagonists and soluble (i.e., trap) receptors attenuate excessive (e.g., disease-associated) TGF-β bioactivity while retaining homeostatic TGF-β signaling functions. Such approaches may avoid the adverse outcomes that result from TGF-β depletion (Yaswen et al., 1996; Yang et al., 2020).

## Multiple Modes of TGF-β1 Activation

The tissue response to injury is largely dependent on multi-level controls on the persistence of TGF-β isoform expression and activation in the immediate pericellular microenvironment. The transition of TGF-β1 from a latent to bioactive configuration is a critical checkpoint in the fibrogenic response. TGF-β1-3 pro-proteins are comprised of a dimeric growth factor and N-terminal latency-associated peptide (LAP) domains. Disulfide bonding between LAP and the latent TGF-β binding protein (LTBP) occurs within the endoplasmic reticulum (Robertson and Rifkin, 2016). In the Golgi, LAP is cleaved from the pro-protein by the subtilisin-like pro-protein convertase furin prior to extracellular transport of the ternary large latent complex, consisting of TGF-β, LAP and the latent TGF-β binding protein (TGF-β/LAP/LTBP). The 4 LTBP isoforms (LTBP1-4) then interact with different structural elements of the ECM including fibrillin microfibrils and the fibronectin network (Zilberberg et al., 2012; Tsuda, 2018). While the different LTBPs exhibit some preferences for TGF-β isoform recognition, LTBP-1 has a particular affinity for fibronectin and, more specifically, for the extra domain A (EDA) splice variant of fibronectin (FnEDA) (Zilberberg et al., 2012; Tsuda, 2018; Zent and Guo, 2018). FnEDA appears particularly critical in TGF-β1 signaling as interference with EDA domain function attenuates both LTBP-1 binding and TGF-β1 activation (Klingberg et al., 2018). Latency, however, is strictly dependent on LAP as a LAP mutant that cannot bind the LTBP effectively retains TGF-β1 in an inactive configuration (Robertson and Rifkin, 2016).

**FIGURE 9:**
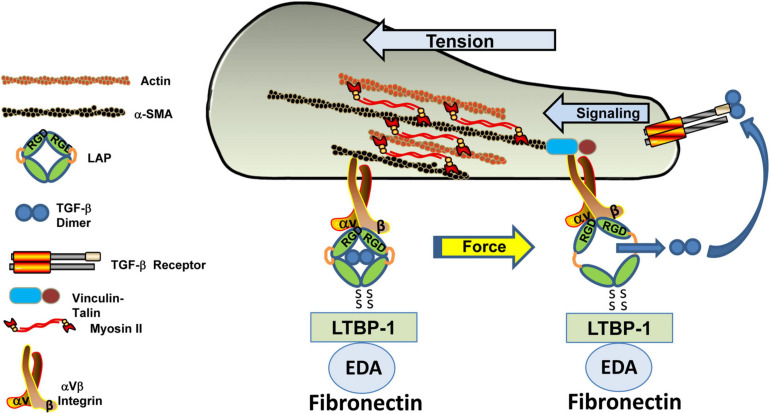
Tension-dependent release of active TGF-β from the LAP cage. The ternary large latent (LTBP/TGF-β/LAP) complex forms a bridge between an αV integrin bound to the RGD site on the latency-associated peptide and LTBP-1 tethered to the fibronectin-rich ECM. Actinomyosin-based contractility generates mechanical tension within this ternary complex inducing a conformational change in the LAP that releases the now-active TGF-β dimer that, in turn, occupies the TGF-βR to initiate downstream signaling.

Mechanisms underlying release of latent TGF-β1 from the LAP cage include proteases, integrins, other proteins such as thrombospondin-1 and various physicochemical factors both alone and in combination (Robertson and Rifkin, 2016). Several proteases cleave the hinge region in LAP freeing the TGF-β dimer for receptor occupancy, although the physiologic relevance of protease-only liberation is complicated by the considerable redundancy in the various participating enzyme systems. Non-proteolytic as well as protease-requiring mechanisms involving αv subunit integrins (e.g., αvβ1, β3, β5, β6, β8), however, also activate TGF-β1 particularly in the context of a progressively fibrosing, increasingly stiff, renal microenvironment (Hysi and Yuen, 2020). Binding of αv integrins to the LAP N-terminal arginine-glycine-aspartic acid (RGD) motif generates Rho/RhoA-dependent tractional forces with ECM-anchored LTBPs; the resulting distortion of the LAP cage liberates and, thereby, activates the TGF-β1 dimer (Buscemi et al., 2011; Hinz, 2015; Sheppard, 2015; Robertson and Rifkin, 2016; Dong et al., 2017; Nickel et al., 2018; Figure 9). Cooperative involvement of both integrins and proteases is an additionally proposed mechanism. One model suggests that tensional strain generated by complex formation between αv integrins and the RGD motif on ECM-tethered LAP predisposes LAP to cleavage by cell surface-proximal proteases (Robertson and Rifkin, 2016). There appears to be, however, significant differences in the type of strain, the activation of latent TGF-β1 and the amplitude of expression of the engaged genes. Compared to steady-state shear strain, oscillatory forces generate significantly greater levels of active TGF-β1 resulting in the increased expression of the profibrotic triad PAI-1, collagen 1A1 and periostin (Kouzbari et al., 2019). Among the αv integrin subtypes, αvβ6 is a major TGF-β1 release trigger and a likely fibrotic effector since renal obstruction in β6-deficient mice is associated with a reduction in TGF-β1 activity and decreases in collagen I, collagen III and PAI-1 expression (Ma et al., 2003). Regardless of the actual pathway, computational modeling suggests that a protease (i.e., plasmin)-dependent bistability mechanism regulates TGF-β1 bioactivity (Li et al., 2017). It appears that TGF-β1 undergoes a bistable switch in response to increasing concentrations of plasmin from a high-level thrombospondin-1-mediated to lower-level predominantly plasmin-dependent mode of activation; both have implications to the development and progression of fibrotic disorders.

αvβ8 also releases TGF-β1 from the LAP:TGF-β1 complex bound to GARP (glycoprotein A repetitious predominant) on the surface of regulatory T cells (Lienart et al., 2018). This mechanism is unique to T-regs; membrane tethered GARP/LAP/TGF-β1 promotes presentation of the LAP RGD sequence to the αvβ8 integrin on adjacent cells. Tensional strain releases and activates the TGF-β1 dimer in much the same way as occurs via ECM-anchored LTBP-1. In addition, recent findings using cryo-electron microscopy to probe LAP:TGF-β complex interactions with the αvβ8 integrin suggests an alternative mode of TGF-β activation that does not necessitate release of dimeric TGF-β from the LAP (Campbell et al., 2020). While the existence of multiple mechanisms of TGF-β1 activation may be cell- and tissue-type dependent, complicating the adaption of a universal therapeutic strategy, pharmacologic inhibition of RGD-binding integrins attenuates renal fibrosis and improves organ function following injury (Basta et al., 2020) and antibody targeting of αvβ6 mitigates bleomycin-induced lung fibrosis (Horan et al., 2008). Integrin-focused therapies, however, are not without controversy. Phase 2 clinical trials of antibody BG00011 (previously known as STX-100), which targets αvβ6 was terminated by Biogen due to safety concerns (Freeberg et al., 2021)^4^.

Elevated TGF-β1 levels, coupled with loss of tissue elasticity, further increases FnEDA expression while promoting LTBP-1/FnEDA co-localization, facilitating integrin/LAP engagement and the subsequent creation of tensional strain stimulating the generation of bioactive TGF-β1 (Wynn and Ramalingam, 2012; Chang et al., 2017; Freeberg et al., 2021). Progressive ECM stiffness and a TGF-β1-rich microenvironment promotes myofibroblast differentiation and survival while activating the Hippo pathway mechanosensitive transcriptional co-activators YAP and TAZ (Liu et al., 2015; Dupont, 2016; Jorgenson et al., 2017; Misra and Irvine, 2018; Santos and Lagares, 2018; Totaro et al., 2018). Convergence of YAP/TAZ and TGF-β1 pathways, in the context of recurrent or persistent tissue injury, induces expression of several major profibrotic genes including CCN2, fibronectin and PAI-1 contributing, thereby, to the eventual development of fibrotic disease (Kim et al., 2019; Figure 7). These findings suggest a complex mechanism for TGF-β1 involvement over the course of renal fibrosis in which induction of FnEDA is a critical element in a TGF-β/FnEDA/αv integrin positive feed-forward loop. It should be mentioned that there have been some attempts to assess these requirements for TGF-β1 mobilization in a translational context. Systemic injection of a bi-specific antibody with FnEDA binding and TGF-β1 neutralizing domains confirmed both construct accumulation and reduced fibrosis in the injured kidney providing supporting evidence for such a model (McGaraughty et al., 2017). How such a strategy may be adapted for patient treatment, however, remains to be determined.

## Involvement of P53 in TGF-β1-Induced Renal Fibrosis

Since TGF-β1 signaling is a major driver of UUO-induced renal fibrosis (Richards et al., 2018), clarification of the involved intermediates downstream of the activated TGF-β receptors may have therapeutic implications for patients with UPJ disease. In the canonical pathway, occupancy of a type II receptor (TGF-βRII) by the TGF-β1 dimer drives complex formation with, and subsequent phosphorylation of, the ALK5 type I receptor (TGF-βRI) that, in turn, phosphorylates receptor (R) SMADs (predominately SMAD2/3 in fibrotic disease) at the distal C-terminal SxS motif (Ser^423/425^ and Ser^465,467^ for SMAD3 and SMAD2, respectively) (Matsuzaki, 2013). While early models suggested that SMAD2 interacts with the SMAD binding domain (SBD) of the SMAD anchor for receptor activation (SARA) followed by SARA:SMAD2 delivery to the TGF-βRI via the C-terminal domain of SARA to facilitate R-SMAD phosphorylation, the actual involvement of SARA in TGF-β signaling is controversial (Rozés-Salvador et al., 2018, 2020). Regardless of the precise mechanism, pR-SMADs complex with the shuttle SMAD4 and translocate to the nucleus to impact transcription of a rather large slate of TGF-β1 responsive genes (Massagué, 2000; Massague, 2012). Identification of differentially expressed genes (DEG), using an unbiased microarray analysis, at two time points post-UUO disclosed 606 upregulated (including 430 annotated) and 485 downregulated (including 251 annotated) genes (Higgins et al., 2003). More than 70 such DEG partitioned to the ECM/cytoskeletal cluster indicative of the breath of targets that may well impact the fibrogenic phenotype. KEGG analysis of the transcriptome of diabetic and non-diabetic mice indicated, in fact, that significant differentially-expressed genes closely associate with the p53 signaling network, as well as the MAPK and TGF-β pathways (Wang et al., 2016).

The growing number of non-canonical (i.e., non-SMAD) elements and their associated pathways in the TGF-β1 network, however, suggests a more significant level of mechanistic diversity in the control of gene expression and the potential existence of an expanding repertoire of regulated sequences (Zhang, 2017). Appropriately recognized as the master regulator of fibrosis (Meng et al., 2016; Lodyga and Hinz, 2020), the TGF-β1 signaling apparatus, including the downstream SMAD effectors, cross-talk with an extensive and highly interactive system that includes the Raf/MEK/ERK, JAK/STAT, Wnt, Notch, Hippo/YAP/TAZ, PI3K/AKT, GSK3/Twist/FOXO, and PKC/Smurf1/RhoA/Rock cascades (Böttinger and Bitzer, 2002; Piersma et al., 2015; Zhang, 2017; Ahmadi et al., 2019; Patel et al., 2019; Finnson et al., 2020; Labibi et al., 2020). The increasing complexity of participating co-factors in the regulation of TGF-β1-responsive genes likely reflects the comparatively low affinity of DNA-SMAD interactions.

One such important co-activator is the tumor-suppressor p53. The involvement of p53 in renal disease was initially defined in a rat model of ischemia-reperfusion injury (Kelly et al., 2003). p53 induction and increased p53 serine 15 phosphorylation is also evident in the kidney following nephrotoxin (e.g., cisplatin, aristolochic acid) administration or UUO, particularly in the dysmorphic epithelium (Zhou et al., 2010; Wei et al., 2007; Samarakoon et al., 2013a,b), and renal allograft rejection (Higgins et al., 2019). Recent studies, furthermore, link tubular epithelial dysfunction in response to both acute (e.g., ischemia-reperfusion, nephrotoxins) and more protracted (UUO) injury to the progression of renal fibrosis via the p53 and JNK pathways with the retention of TGF-β signaling (Yang et al., 2010). p53 is activated in the injured renal epithelium initiating cell cycle arrest at the G_1_ and G_2_/M checkpoints depending on the participating effectors (e.g., ATM, ATR, CK1, CK2, p21, TGF-β1) and extent of tissue hypoxia (Thomasova and Anders, 2015; Tang et al., 2018, 2019, 2020; Liu et al., 2019). TGF-β1 signaling in the damaged kidney increases p53 levels and phosphorylation, particularly at p53^*S*9/15^, promoting p53 stabilization and triggering p53-SMAD2/3 interactions resulting in transcription of the growth inhibitor p21 and subsequent p21-dependent G_1_ arrest (Higgins et al., 2019). While p21 is a major p53 responsive gene, p53 upregulation in hypoxic tubular cells also suppresses CDK1, cyclin B_1_, and cyclin D_1_ expression, potentially increasing residence time in G_2_/M. Such interrelationships are complicated, however. Oscillations in p53/p21 transcription impact accumulation of p21 protein and, thereby, cellular arrest and death programs. Single-cell analysis indicates that p21 transcription reflects p53 dynamics although p21 protein levels increase only gradually (Hafner et al., 2020).

Molecular approaches confirmed the involvement of p53 in several models of injury-induced kidney disease. While p53^–/–^ mice largely retain renal architecture and function following cisplatin or aristolochic acid treatment, wild-type animals develop severe renal damage exhibiting all the hallmarks of a maladaptive repair process (Wei et al., 2007; Zhou et al., 2010). siRNA-directed silencing of p53, moreover, mitigates the severity of cisplatin- and ischemic-induced kidney damage (Molitoris et al., 2009). Pharmacologic inhibition of p53 activation with pifithrin-α, delivery of p53 siRNA or genetic deletion of p53 in the proximal tubular epithelium attenuates both prolonged G_2_/M residence and the fibrotic response to cisplatin, UUO or ischemic injury (Wei et al., 2007; Yang et al., 2010; Ying et al., 2014; Tang et al., 2018; Higgins et al., 2019; Liu et al., 2019; Molitoris, 2019). Similarly, cisplatin- or bilateral ischemia-induced AKI in streptozotocin-treated mice or genetically susceptible (Akita) diabetic animals is significantly diminished by pifithrin-α, p53 siRNA or proximal tubule-targeted p53 ablation (Peng et al., 2015). There appears to be a timing dependency, however, for maximal efficiency with short term p53 knockdown (i.e., day 14) effective at reducing both the senescence cellular load and the ischemic phenotype; a longer course of p53 siRNA administration did not provide any additional therapeutic benefit (Baisantry et al., 2019).

Assessment of the toxicologic and pharmacokinetic properties of 2′-O-methyl sugar-modified p53 siRNAs indicated preferential localization to, and rapid uptake (peak levels 5–30 min post-inoculation) by, the kidney as well as short residence duration in the proximal tubular epithelium (Thompson et al., 2012). Most encouraging from a potential clinical utility perspective, p53 knockdown was achieved within 3–6 h after intravenous administration of a single bolus of 12 mg/kg of these modified siRNAs to animals with ischemic- hypoperfusion- and cisplatin-induced renal injury (Molitoris et al., 2009) which is well below the dose of 200 mg/kg that corresponded to the no observable adverse effect level (NOAEL) in the rat (Thompson et al., 2012). Intravenously-delivered p53 siRNA, moreover, also mitigates the structural and functional damage to transplanted kidneys upon ischemia/reperfusion injury in two syngeneic rat models (Imamura et al., 2010), consistent with 2′-O-methyl sugar-modified siRNA knockdown of p53 transcripts (Molitoris et al., 2009), suggesting the potential clinical utility of targeting p53 in patients with failing renal allografts.

## Genomic Targets of TGF-β1/P53 Signaling in Obstructive Renal Disease

TGF-β1 stimulates p53 transcriptional activity largely by serine phosphorylation in the N-terminus transactivation domain and serine/lysine acetylation/methylation in the C-terminal tetramerization and regulatory domains (Maclaine and Hupp, 2009) facilitating interactions between p53 and SMAD2/3 (Piccolo, 2008; Overstreet et al., 2014). Phosphorylated p53 and SMAD2/3 form transcriptionally active multi-protein complexes on the promoter regions of a subset of TGF-β1 target genes (Cordenonsi et al., 2003, 2007; Piccolo, 2008; Overstreet et al., 2014, 2015). Such reprogramming may not be reflected, however, in detectable changes in p53 protein abundance. In this regard, short-term treatment with Nutlin-3, which interferes with the p53-binding hydrophobic pocket in MDM2 functioning thereby as a p53 competitive inhibitor, results in p53-dependent transcription of a large complement of direct target genes without any significant increase in p53 levels, at least in the brief window of Nutlin-3 exposure used (Allen et al., 2014). While these findings suggest that the expression of novel, albeit likely low abundance, genes may be independent of perceived changes in p53 cellular abundance, p53 pulsing (i.e., stimulus-dependent changes in the amplitude, duration and period of p53 levels) impacts TGF-β1-response gene dynamics differently than transcription patterns evident under gradually increasing p53 levels (Porter et al., 2016). Indeed, single cell transcriptome profiling revealed that mitigating p53 pulsing by retaining p53 at high levels by treatment with Nutlin-3 results in the creation of a single, large network of coordinated genes rather than the two discrete subnetworks evident under conditions that allow p53 oscillation (Porter et al., 2016).

**FIGURE 10:**
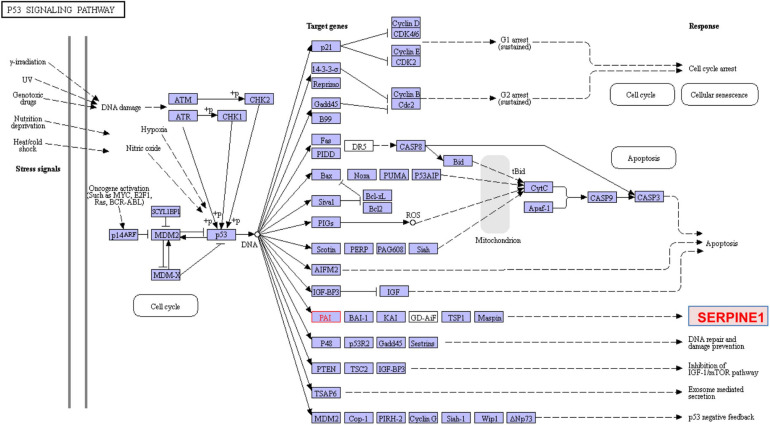
KEGG p53 signaling. p53 is mobilized in response to various signals including DNA damage, oxidative stress and oncogene activation usually by phosphorylation by elements of the ATM/ATR pathway. p-p53 initiates transcription of target genes that result in cell cycle arrest (at both G_1_ and G_2_/M), acquisition of a senescent phenotype or apoptosis. SERPINE1 (PAI-1) and p21 are prominent members of both the cellular senescence (Figure 6) and p53 signaling pathways. TGF-β1 also activates p53 stimulating p53^Ser9,15^ phosphorylation and acetylation, promoting interactions with activated SMADs and subsequent binding of p53/SMAD3 complexes to the PAI-1 promoter in human renal tubular epithelial cells (Overstreet et al., 2014). Consistent with a potential *in vivo* role for p53 and SMADs in TGF-β1-driven renal fibrosis, co-induction of pSMAD2/3, p53, p53^Ser15^ and the target growth arrest/senescence genes SERPINE1 and p21 are evident in the tubular epithelium of the obstructed kidney (e.g., Overstreet et al., 2014; Patel et al., 2019).

**FIGURE 11:**
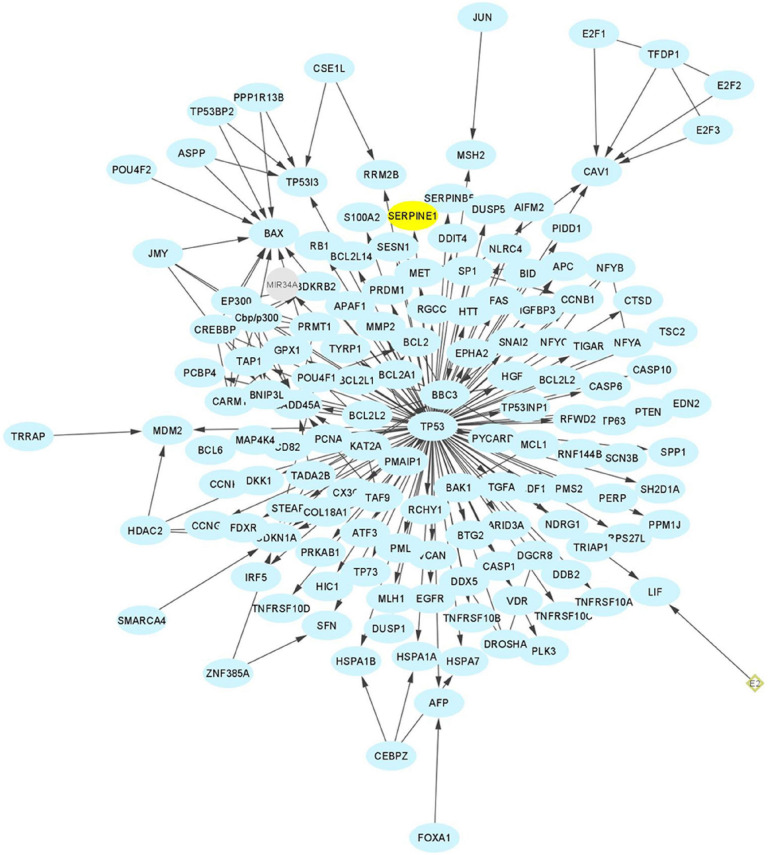
Direct p53 effectors. There are a significant number of p53 target genes that control a broad range of cellular processes, including cell cycle arrest, cell senescence, DNA repair, metabolic adaptation and cell death. Of these, approximately 135 are listed in the Pathway Interaction Database as direct p53 effectors. SERPINE1 (PAI-1) (highlighted in yellow) is among the most prominent since, as a major p53 target gene, it regulates cell proliferation and migration, ECM remodeling, wound healing, invasion and metastasis, senescence and survival (e.g., Higgins et al., 2018, 2019; Tang et al., 2018).

The KEGG-defined p53 signaling pathway and the cooperative p53/TGF-β1 genomic cluster, moreover, includes genes involved in cell growth control and ECM remodeling (Dupont et al., 2004; Elston and Inman, 2012; Slattery et al., 2019; Figure 10). While the molecular basis for this co-dependency requires clarification, many TGF-β1-responsive sequences possess p53-recognition motifs as well as SMAD-binding elements (Wang et al., 2001; Takebayashi-Suzuki et al., 2003; Allen et al., 2005; Qi et al., 2006). Indeed, p53 participates in the transcription of several renal disease-causative genes including CNN2, collagen I and SERPINE1 (PAI-1) underscoring the complexities of non-canonical pathways in TGF-β1-induced fibrosis (Kodama et al., 2011; Elston and Inman, 2012; Overstreet et al., 2014, 2015). PAI-1 is a member of the “high-confidence” complement of 151 p53 target genes (Chang et al., 2014), a major p53 direct effector hub gene (Figure 11) and ranks 131 among 343 of the most prominently identified p53-activated genes (Fischer, 2017). In view of the extensive repertoire of direct (i.e., 943 genes; the p53 cistome) and indirect p53-responsive genes, stringent criteria are required for identification of actual p53 genomic targets (e.g., Ali et al., 2014; Nguyen et al., 2018) (summarized for PAI-1 in Table 1). The two 10-bp p53 response elements (p53-REs) in the PAI-1 promoter, which provide a platform for p53 docking in a typical dimer-of-dimers configuration, moreover, are not separated by a nucleotide spacer (Kunz et al., 1995; Riley et al., 2008; Fischer, 2017). Variable p53-RE spacing affects transactivation of target genes. Indeed, *in vivo* analysis disclosed that decamer pairs with no spacers exhibited a strong preference of p53 binding (Nguyen et al., 2018). This has pathophysiologic implications since p53-RE penalty scoring indicates that a high negative impact was attributed to spacers longer than two nucleotides and that p53-REs with long spacer length are not likely to be bound by p53 *in vivo* (Tebaldi et al., 2015).

**TABLE 1:** Summary of p53 involvement in expression of the target PAI-1 gene.

1. TGF-β1 stimulates p53^ser9/15^ phosphorylation promoting binding of p-p53/SMAD3 complexes to the PAI-1 promoter in HK-2 human renal tubular epithelial cells (Overstreet et al., 2014).
2. Two canonical p53-binding motifs in the PAI-1 promoter (Kunz et al., 1995; Parra et al., 2001; Riley et al., 2008) meet the >90 p53MH score threshold required for the identification of potential p53-responsive genes (Hoh et al., 2002). Both p53-binding sites (nt −224 to −204) are proximal to the transcription start site.
3. PAI-1 is a member of the “high confidence” cohort of 151 p53 target genes (Chang et al., 2014).
4. PAI-1 ranks 131 among 343 of the most prominent p53-activated genes (Fischer, 2017).
5. PAI-1 is included in the p53 Target Database (Fischer, 2017).
6. PAI-1 is a component in the p53 circuit board (Sullivan et al., 2012).
7. p53 silencing, genetic ablation/subsequent rescue, and pharmacological inhibition confirmed that p53 is required for PAI-1 expression in TGF-β1-stimulated cells (Overstreet et al., 2014).
8. TGF-β1-initiated PAI-1 expression is attenuated in p53 knockdown cells (Cordenonsi et al., 2003).
9. p53^–/–^ fibroblasts are not inducible for increased PAI-1 expression in response to TGF-β1 (Samarakoon et al., 2013a,b).
10. Pretreatment of Mv-1Lu mink lung cells (stably expressing a PAI-1 promoter-luciferase reporter construct) with the p53 inhibitor pifithrin-α effectively suppressed TGF-β1-dependent PAI-1 transcription (Samarakoon et al., 2013a,b).
11. Radiation-induced PAI-1 expression requires p53/SMAD3 cooperativity (Milliat et al., 2008).
12. Mutation of the p53-binding sites in the PAI-1 promoter inhibits γ-radiation-induced PAI-1 transcription and attenuates the dual γ-radiation + TGF-β1 synergy (Hageman et al., 2005).
13. γ-radiation did not induce PAI-1 expression in p53-null cells; p53 expression rescue largely restored the PAI-1 response to γ-radiation + TGF-β1 co-stimulation (Hageman et al., 2005).
14. p53 knockdown mitigates radiation-dependent PAI-1 expression (Szołtysek et al., 2018).
15. Overexpression of the Δ133p53α isoform of p53, which lacks the two transactivation domains, inhibits expression of the p53-inducible genes p21, IGFBP7, and PAI-1 (Fujita, 2019).

GenBank annotation and computational analysis identified at least one p53 binding motif within 2,000 bp upstream of the transcription start site in approximately 1,100 human genes; transcript mapping added significantly to the number of candidate p53-regulated promoter sequences (Wang et al., 2001). Alignment of the complement of differentially expressed TGF-β1-induced genes (DEGs) with the p53 Target Database further revealed that the majority of those responsive to TGF-β also possesses p53 binding sites and recent studies clarified the transcriptional basis for such TGF-β1/p53 cross-talk (Elston and Inman, 2012; Overstreet et al., 2014; Kawarada et al., 2016; Higgins et al., 2018). Among the impacted set of DEGs, which includes the fibrosis-causative PAI-1 and CNN2 genes, pharmacologic inhibition of p53 function may well have clinical implications in disease management. Indeed, p53 is required for TGF-β1-induced expression of fibronectin, CNN2 and PAI-1, the latter a major profibrotic hub gene and critical factor in the initiation as well as progression of TGF-β1-dependent fibrotic disease (Higgins et al., 2018; Xu et al., 2020).

TGF-β1 impacts p53 function, and thereby the fibrotic program, via an additional (albeit complex) mechanism that involves the serum response factor (SRF) and its co-activators including the myocardin-related transcription factors (MRTF-A, MRTF-B) and the ternary complex factors (TCF) (Small, 2012; Gau and Roy, 2018; Onuh and Qiu, 2021). This pathway is likely to be particularly important in the TGF-β1-directed differentiation of interstitially-trafficked vascular pericytes into the pro-fibrotic, highly contractile and α-smooth muscle actin-rich myofibroblast lineage. Indeed, complex formation between SRF and MRTF vs. TCF dictates the particular subset of SRF target genes induced (Onuh and Qiu, 2021). Emergence of the myofibroblastic phenotype appears coupled to joint regulation by the TGF-β1 and MRTF-A/SRF signaling networks which cooperate to promote expression of a distinct set of contractile and profibrotic genes (Olson and Nordheim, 2010; Small, 2012; Velasquez et al., 2013; Gau and Roy, 2018; Werner et al., 2019). Altered actin cytoskeletal dynamics, as a function of growth factor stimulation or a changing mechanical environment, releases MRTF-A from its G-actin cytoplasmic tethers facilitating MRTF-A nuclear translocation and interaction with SRF to trigger transcription of SRF target genes which possess the CArG consensus element (e.g., collagen I, α-smooth muscle actin) many of which are also TGF-β1/p53 responsive genes as well. It was noted in recent reports (Werner et al., 2019; Onuh and Qiu, 2021), in fact, that there appears to be appreciable overlap in the TGF-β1/p53 and Rho/MRTF/SRF genomic signatures (e.g., Esnault et al., 2014) consistent with the role of MRTF-A in myofibroblastic differentiation (Crider et al., 2011). Interestingly, SRF/TCF complexes appear to drive the increased transcription of MDM4 (Pellegrino et al., 2021), a key member of the MDM2/MDMX/MDM4 repertoire of p53 activity regulators. Collectively, these findings indicate that there is significant context-specific cross-talk between the TGF-β1/SMAD3/p53 and Rho/MRTF/TCF/SRF signaling pathways that impact the expression of gene clusters that regulate the adaptive and maladaptive tissue repair outcomes. The differential partnering of SRF with MRTF vs. TCF may well determine, at least in part, the particular subset of SRF-dependent genes engaged and the nature of the wound healing response.

## Conclusion

PAI-1 negatively regulates the plasmin-dependent pericellular proteolytic cascade effectively limiting ECM degradation and fibrinolytic activity contributing, thereby, to the initiation and/or progression of interstitial fibrosis and progressive renal disease (Ghosh and Vaughan, 2012; Flevaris and Vaughan, 2017; Figure 3). PAI-1 deficient mice are, in fact, protected from excessive ECM accumulation in several organ systems including the kidney and PAI-1 decoys attenuate both UUO-initiated and established interstitial fibrosis (Gonzalez et al., 2009). Apart from an impact on ECM turnover, the p53→PAI-1 axis likely drives renal cell stalling in both G_1_ and G_2_/M (Kelly et al., 2003, 2013; Kortlever et al., 2006; Yang et al., 2010; Overstreet et al., 2014; Moonen et al., 2018; Wu and Prives, 2018; Liu et al., 2019; Oliva-VIlarnau et al., 2020) suggesting an additional mechanism for the repair deficiency. PAI-1 is a prominent member of both the growth arrest/fibrosis genomic cluster in the diabetic rat kidney (Kelly et al., 2013) and the 11-gene urine mRNA signature predictive of human renal allograft fibrosis (Anglicheau et al., 2012). While initially a protective response, when trauma is chronic or sustained, the associated G_2_/M arrest contributes to development of kidney disease due to TGF-β1-directed expression of profibrotic factors (Canaud et al., 2019; Koyano et al., 2019). During the initial stages of UUO-induced renal damage or ischemia/reperfusion injury, tubular cells arrest in G_2_ due, albeit perhaps partially, by a p21-dependent pathway (Yang et al., 2010; Lovisa et al., 2015; Canaud et al., 2019; Koyano et al., 2019).

Similar to p53 deficiency, PAI-1 knockdown also results in escape from TGF-β1-induced cytostasis in various cell types including those derived from the renal proximal tubular epithelium (Kortlever et al., 2008; Overstreet et al., 2015). MEFs from PAI-1^–/–^ mice proliferate well beyond the senescence checkpoint while ectopic expression of PAI-1 in p53-null fibroblasts rescues a phenotype displaying the hallmarks of replicative senescence-induced growth inhibition (Kortlever et al., 2006). PAI-1 expression in response to TGF-β1 is required for a senescence-associated proliferative arrest, moreover, and PAI-1-deficient mouse embryonic fibroblasts (MEFs) or PAI-1 knockdown in wild-type MEFs and human keratinocytes confers resistance to TGF-β1-induced growth arrest (Kortlever et al., 2006, 2008). Indeed, human keratinocytes engineered to overexpress PAI-1 enter into growth arrest while overexpression of PAI-1 alone is sufficient to halt G_2_/M transit in proximal tubule cells (Kortlever et al., 2008; Gifford et al., 2021). Cellular PAI-1 “status” has a profound effect on genomic reprogramming as transcript profiling indicates that 1,283 genes are upregulated in PAI-1 knockdown cells while 1,891 are reduced suggesting that PAI-1 negatively and positively impacts gene expression, either directly or indirectly. Among the genes repressed by PAI-1 deficiency are members of the SASP complement, a finding consistent with the involvement of YAP-induced PAI-1 as a major contributor to an oncogene-induced senescent phenotype (Marquard et al., 2020). Since renal repair requires a regenerative phase to replace injured or dying tubular epithelial cells, incomplete injury resolution compromises nephron function and leads to persistent inflammation and increased matrix deposition. The prolonged G_1_ and G_2_/M arrest in severe AKI, although necessary to insure DNA fidelity and maintain genome integrity, may adversely impact regenerative growth if cell cycle re-entry is excessively delayed while promoting maladaptive fibrotic repair in a TGF-β1-rich environment (Yang et al., 2010; Moonen et al., 2018).

Collectively, it appears that p53 plays an important role in TGF-β-induced proliferative arrest via induction of both p21 and PAI-1 transcription and that loss of p53 or its target gene PAI-1 confers resistance to TGF-β1-mediated growth inhibition. The mechanism remains to be clarified but recombinant PAI-1 induces collagen I and fibronectin expression in renal mesangial cells via a TGF-β1-dependent mechanism and PAI-1 stimulates TGF-β1 promoter activity (Seo et al., 2009). PAI-1 may initiate, perhaps maintain, a pro-fibrogenic “loop” in the context of renal disease (Nicholas et al., 2005; Seo et al., 2009). It is tempting to speculate, therefore, that targeted down-modulation of PAI-1 expression or function may provide multi-level therapeutic opportunities to inhibit the onset and progression of tissue fibrosis.

## Author Contributions

CH, JT, SH, CG, and AC performed the experiments. RS and PH designed the experiments and analyzed the data. BM, DJ, WZ, DC, RS, and PH provided pathology consultation and wrote the manuscript. All authors contributed to the article and approved the submitted version.

## Conflict of Interest

The authors declare that the research was conducted in the absence of any commercial or financial relationships that could be construed as a potential conflict of interest.

## Footnotes

1 nccd.cdc.gov/ckd

2 www.healthdata.org/gbd

3 www.nikkd.nih.gov/health-information/kidneydisease

4 www.fiercebiotech.com

## Abbreviations

AQP2: aquaporin 2
ALK: activin-like kinase
AKI: acute kidney injury
ATM: ataxia telangiectasia mutated
ATR: ataxia telangiectasia and Rad3-related serine/threonine-protein kinase
CASP: chronic kidney disease-associated secretory phenotype
CHK1: checkpoint kinase 1
CHK2: checkpoint kinase 2
CKD: chronic kidney disease
CTGF: connective tissue growth factor
DEG: differentially expressed genes
Dot1l: disruptor of telomeric silencing-1-like
ECM: extracellular matrix
EDA: extra domain A
ESRD: end stage renal disease
FnEDA: extra domain A splice variant of fibronectin
GARP: glycoprotein A repitious predominant
KIM-1: kidney injury molecule-1
LAP: latency-associated peptide
LTBP: latent transforming growth factor- β 1 binding protein
MRTF: myocardin-related transcription factors
PAI-1: plasminogen activator inhibitor-1
PIF-alpha: pifithrin- α
RGD: arginine-glycine-aspartic acid
SASP: senescence-associated secretory phenotype
SERPIN: serine protease inhibitor
SERPINE1: serine protease inhibitor, clade E, member 1
siRNA: small interfering RNA
SRF: serum response factor
TASCC: target or rapamycin-autophagy spatial coupling components
TCF: ternary complex factors
TGF- α 1: transforming growth factor- β 1
TGF- β R: transforming growth factor- β receptor
TAZ: transcriptional coactivator with PDZ-binding motif
UPJ: ureteropelvic junction
UUO: unilateral ureteral obstruction
YAP: yes-associated protein.
